# A novel fusogenic herpes simplex virus for oncolytic virotherapy of squamous cell carcinoma

**DOI:** 10.1186/1743-422X-8-294

**Published:** 2011-06-10

**Authors:** Hiroo Takaoka, Gen Takahashi, Fumi Ogawa, Tomoaki Imai, Soichi Iwai, Yoshiaki Yura

**Affiliations:** 1 Department of Oral and Maxillofacial Surgery, Osaka University Graduate School of Dentistry, Osaka University, Suita, Osaka, Japan

**Keywords:** herpes simplex virus mutant, oncolytic virotherapy, oral squamous cell carcinoma, cell fusion

## Abstract

**Background:**

R849 is a neurovirulent γ_1_34.5 gene-deficient form of herpes simplex virus type 1 (HSV-1) and has LacZ genes at the deleted sites of the γ_1_34.5 gene. HF is a spontaneously occurring, fusogenic HSV-1 strain. The purpose of this work was to generate a virus that has the syncytial character of HF, while preserving the γ_1_34.5 gene inactivation profile of R849 virus.

**Results:**

Vero cells were infected with R849 and HF simultaneously and two viruses, RH1 and RH2, expressing the LacZ gene and inducing extensive cell fusion were selected. A polymerase chain reaction (PCR)-based analysis suggested that one copy of the γ_1_34.5 gene is lost in RH1, whereas both copies are lost in RH2, and that the γ_1_34.5 gene is replaced by a R849-derived DNA fragment with the LacZ gene. These viruses produced larger plaques and more progeny than the parental viruses. Infection with RH2 decreased the viability of oral squamous cell carcinoma (SCC) cells most strongly. When RH2 was injected into xenografts of oral SCC in nude mice, multinucleated cells were produced and the growth of the tumors was suppressed significantly.

**Conclusion:**

These results indicate that novel oncolytic HSV-1 vectors can be produced with the genetic background of the oncolytic HSV-1 HF, and that RH2 is deficient in γ_1_34.5 genes and shows extensive cytopathic effects in oral SCC cells. RH2 may be useful in oncolytic virotherapy for oral SCC.

## Background

Carcinoma of the oral cavity represents 4% of all malignancies in men and 2% in women, with the majority of these tumors being squamous cell carcinomas (SCCs)[1]. Despite improvements in both the surgical and pharmacological management of oral malignancies, the 5-year survival rate for oral cancer is very poor at approximately 53% mainly because of the advanced state of the disease at the time of presentation [2]. There is no effective treatment for advanced symptoms such as skin metastasis.

Oncolytic virotherapy with herpes simplex virus type 1 (HSV-1) is based on the ability of an attenuated virus to destroy the infected tumor cells [3-5]. Most cancer patients have immunity against HSV-1 and require repeated administration of the HSV-1 vector, so the virus must be delivered locally to prevent its inactivation by neutralizing antibodies [6-8]. In this aspect, oral cancer has the advantage of accessibility.

A number of HSV-1 vectors have been developed for solid tumors. Most of these have the main neurovirulence gene γ_1_34.5 removed, which severely restricts their ability to replicate in the adult central nervous system and cause a latent infection [9,10]. G207 has two deletions at γ_1_34.5 and ribonucleotide reductase (UL39)[11]. NV1020 expresses one of the two γ_1_34.5 genes allowing the virus to replicate more efficiently without compromising safety [6,12,13]. OncoVEX^GM-CSF ^is deleted of the γ_1_34.5 gene, and ICP47, which otherwise blocks antigen presentation. In addition, a granulocyte macrophage colony-stimulating factor (GM-CSF) gene is inserted to enhance the immune response to tumor antigens released after the virus replicates [7]. Another type of HSV-1 vector developed to improve local antitumor activity has the ability to form a syncytium [8,14]. HF10, a clone of HF, is a spontaneously occurring, highly attenuated virus [15-17]. It too can form a large syncytium in a variety of cell types and has strong anti-tumor activity. In clinical trials, HF10 was found to be effective against breast cancer, pancreatic cancer, and head and neck cancer [18-20].

We reported that a combination of γ_1_34.5 gene-deficient HSV-1 R849 [21] and HF exerted a greater suppressive effect on xenografts of human oral SCC in nude mice than did repeated injections of R849 [22], indicating the effectiveness of HF as an oncolytic virus. We speculated that recombinant forms of these viruses might be highly fusogenic (as HF) and still lack the neurovirulent γ_1_34.5 gene. To examine this possibility, an attempt was made to select candidate recombinants after infecting permissive cells with R849 and HF simultaneously. An isolate from the cultures, RH2, induced extensive cell fusion in cell cultures and cancer tissue and suppressed tumor growth at a low dose.

## Materials and methods

### Cells and virus

The human oral SCC cell line SAS was obtained from the Japanese Collection of Research Bioresources (Tokyo, Japan). SAS cells were cultured in Dulbecco's modified Eagle's medium supplemented with 10% fetal bovine serum, 2 mM L-glutamine, 100 U/ml penicillin, and 100 μg/ml streptomycin and grown in an incubator at 37°C in a humidified atmosphere with 5% CO_2_. For Vero monkey kidney cells, Eagle's minimal essential medium containing 5% calf serum and 2 mM L-glutamine was used. HSV-1 mutant R849 [22,23] and HF [15,16] were grown in semi-confluent Vero cell monolayers. The infectivity of HSV-1 was determined by plaque formation on Vero cell monolayers covered with 0.3% methylcellulose.

### The 3-(4,5-dimethylthiazol-2-yl)-2,5-diphenyltetrazolium bromide (MTT) assay

Cells grown in 96-well culture dishes were infected with HSV-1 at a multiplicity of infection (MOI) of 0.01, while controls were mock- infected. After incubation for various intervals, 10 μl of a 5 mg/ml MTT solution was added to each well with 100 μl of medium. Cells were allowed to incubate for 4 h at 37°C, and then 100 μl of 0.04 N HCl in isopropanol was added. They were mixed thoroughly to dissolve the dark blue crystal. After standing overnight at room temperature, the plates were read on a Benchmark Plus microplate spectrophotometer (Bio-Rad Laboratories, Hercules, CA) with a reference wavelength of 630 nm and a test wavelength of 570 nm. Background absorbance at 690 nm was subtracted from the 570 nm reading. Changes from controls (room air) were calculated.

### Polymerase chain reaction (PCR) analysis

The γ_1_34.5 and lacZ gene sequences were amplified by PCR using specific primers [23]: LacZ forward, 5'-GCGTTACCCAACTTAATCG -3'; and LacZ reverse, 5'-TGTGAGCGAGTAACAACC -3' (PCR product; 320 bp), γ_1_34.5 forward, 5'-TCGTCGGACGCGGACTCGGGAACGGTGGAG-3' γ_1_34.5 reverse, 5'-CTCCACGCCCAACTCGGAACCCGCGGTCAG-3' (PCR product; 132 bp)

The amplification was carried out in a volume of 50 μl for 30 cycles with a denaturing temperature of 94°C for 30 sec, an annealing temperature of 60°C for 30 sec, and an extension temperature of 72°C for 2 min using a GeneAmp PCR system 9700 (Applied Biosystems, CA, USA). PCR products were subsequently size-fractionated on 2% agarose gels, stained with 1 μg/ml ethidium bromide and photographed under transmitted UV light.

### Digestion of viral DNA and Southern blot analysis

Viral DNA was isolated after the lysis of infected Vero cells with SDS-proteinase K, repeated phenol-chloroform extraction, following the method of Hirt [24], and ethanol precipitation. For Southern blot analysis, DNA digested by Bam HI endonuclease (NEW ENGLND BioLabs, MA, USA) was separated by agarose gel electrophoresis and transferred to a nylon membrane Hybond-N+ (Amersham Biosciences, UK). The PCR product of LacZ was isolated from the gel, subjected to sequencing and used as a probe. Amplified DNA fragment of LacZ was labeled by Gene Images AlkPhos Direct Labeling and Detection System (Amersham Biosciences). After hybridization, bounded probe was detected with CDP-Star Detection reagent (Amersham Biosciences).

### Animal experiments

Athymic 5-week-old BALB/c (nu/nu) female mice were obtained from Clea Japan (Tokyo, Japan). Mice were subcutaneously injected with 1 × 10^6 ^SAS cells. Once the tumor reached approximately 5 mm in diameter, animals were divided into 4 groups of 6 animals each. Animals were administered a single intratumor (i.t.) injection of 1 × 10^5 ^plaque forming units (PFU) of R849, HF, or RH2 suspended in 50 μl of phosphate-buffered saline (PBS ). Animals in the control group received PBS instead of HSV-1. The experiment was started at the time HSV-1 was injected. Bidimensional tumor measurements were performed for 6 weeks with calipers, and tumor volume was determined using the formula for a rotational ellipsoid (L × W^2 ^× 0.5).

To determine the virus yield in the tumor, an experiment with acute HSV-1 infection was performed. Tumor-bearing mice received an i.t. injection of 1 × 10^6 ^PFU of R849, HF, or RH2. The mice were sacrificed at 3, 7, and 14 days after injection of HSV-1 and tumors were removed. They were suspended in two volumes of PBS, homogenized and centrifuged after three rounds of freezing and thawing. The supernatant was harvested and the virus titer (PFU/ tumor (g)) was measured by plaque assay using Vero cell monolayers.

Another series of experiment was performed for immunohistochemical staining. HSV-1 was injected in the tumors at a dose of 1 × 10^6 ^PFUs and the tumors were removed at 3 and 7 days after injection. Tumors that received PBS were used for the control. Experiments were performed with the approval of the Institute of Laboratory Animals, Osaka University Graduate School of Dentistry.

### Histopathological examination and 5-bromo-4-chloro-3-indolyl-β-D- galactopyranoside (X-gal) staining

For X-gal staining, R849- infected cells were placed in a fixative containing 0.2% glutaraldehyde and 2% formaldehyde in PBS for 1 h, and submerged in cold PBS. Cells were then left overnight in a substrate solution containing 1 mg/ml X-gal (Sigma), 5 mM potassium ferricyanide, 5 mM potassium ferrocyanide, and 2 mM magnesium chloride in PBS and washed with PBS.

Tumors were removed, placed in 10% buffered formalin for fixation, and embedded in paraffin wax. Sections were stained with hematoxylin and eosin (H-E). For immunohistochemical staining, endogenous peroxidase was blocked by incubation in 3% H_2_O_2 _in water for 5 min at room temperature. Sections were washed in PBS and then incubated with rabbit polyclonal antibody against HSV-1 (diluted 1:500, DAKO, Glostrup, Denmark) for 30 min at room temperature. After another wash, slides were reacted with Envision+System-HRP Labelled Polymer Anti Rabbit (DAKO) for 30 min at room temperature. HSV-1 antigen was visualized by treating with diaminobenzidine (DAB) (DAKO, Glostrup, Denmark), counterstained with hematoxylin, and mounted with Entellan (Merck, Darmstadt, Germany). Computer-assisted analysis of immunohistochemical staining was performed using WinROOF image-processing software (Mitani Corp., Tokyo, Japan). This software allowed accurate identification and calculation of the immunostained area [25]. The size (μm^2^) of HSV-1 antigen-positive multinucleated cells was calculated after examining signals in 3 to 5 sections, using high-power ( × 40 objective and × 10 ocular) magnification. At least 3 samples were measured and mean values ± standard deviation (SD) were determined.

### Statistical analysis

An SPSS for Windows computer program (SPSS Inc., Chicago, IL, USA) was used for statistical analyses. Results are reported as means ± SD. Comparison of mean cell viability, plaque size and virus yield in tumors were achieved using one-way ANOVA, followed by Tukey's honestly significant differences (HSD) test. Mean size of HSV-1 antigen-positive multinucleated cells was compared using the unpaired *t*-test. For the repeated measures part of the analyses of tumor volumes, general linear model (GLM) procedure was used to conduct a repeated measures design analyzed. When overall analyses determined significance, Tukey's HSD test was used to examine pairwise differences. P values of less than 0.05 were considered statistically significant.

## Results

### Production of recombinant viruses in Vero cells

**Figure 1:**
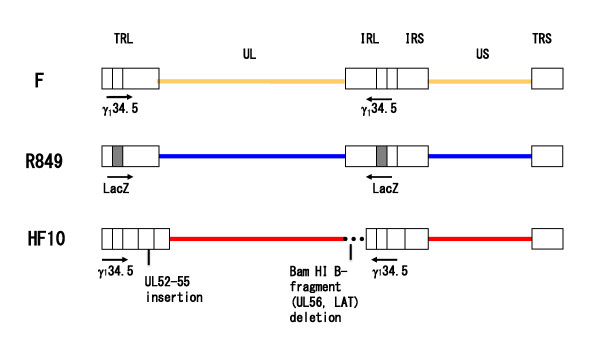
**Schematic presentation of genomic structures of wild-type HSV-1 strain F, γ_1_34.5-deficient HSV-1 R849, and HF10, a clone of HF that is a spontaneously occurring, highly attenuated HSV-1**.

**Figure 2:**
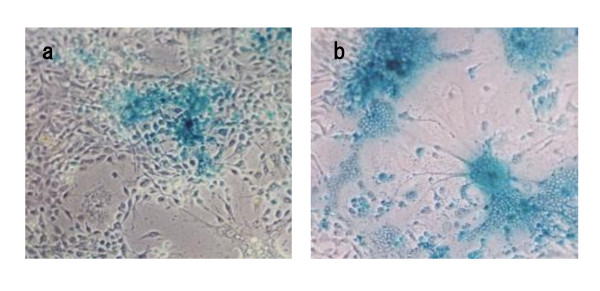
**Cytopathic effect of R849 (a) or RH2 (b) in human oral SCC cells**. SAS cells were infected with HSV-1 at an MOI of 0.01 and cultured at 37°C for 24 h. The infected cells were fixed and subjected to X-gal staining.

HSV-1 R849 has a LacZ gene inserted at the deleted site of the γ_1_34.5 gene [21]. R849-infected cells became rounded and showed X-gal staining, indicating the expression of LacZ. The BamHI profiles of HF10 showed a loss of the B and E fragments and the genome has a deletion of 3832 bp to the right of the UL and UL/IRL junction [17]. Cell-fusion appeared in the cells infected with HF as well as HF10 [22,26]. Schema of the genomic structure of these viruses are shown in Figure 1. In this study, an attempt to generate a virus that has the syncytial character of HF, while preserving the γ_1_34.5 gene inactivation profile of R849 virus by recombination in cell culture was made. For recombination, Vero cells were infected with R849 and HF simultaneously, at different MOIs. The MOI was 0.01 for R849 and changed from 0.001 to 0.1 for HF. When the MOI of HF was 0.02 or 0.03, several syncytia with X-gal staining were observed and syncytia were pick up under a phase contrast microscope. Viruses recovered from the syncytia were diluted and underwent plaque formation in Vero cells. This cloning procedure was repeated at least five times and two virus clones showing extensive cell fusion, RH1 and RH2, were used for further study. When human oral SCC SAS cells were infected with R849, and cultured for 24 h, rounded cells were positive for X-gal staining. Large syncytia were positive for X-gal staining in RH2-infected cells (Figure 2).

### PCR analysis of RH1 and RH2

**Figure 3:**
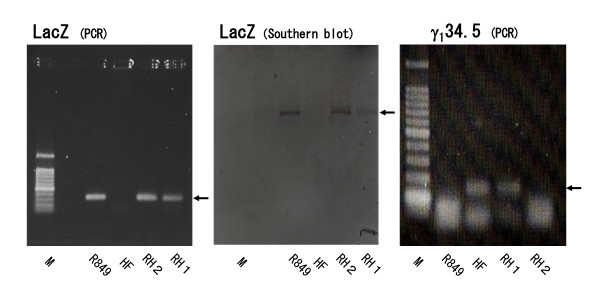
**PCR and Southern blot analyses for LacZ and γ_1_34.5 genes**. PCR analysis was performed using primers for LacZ and γ_1_34.5 genes and DNA of HSV-1-infected cells. The PCR products were separated by agarose gel electrophoresis and stained by ethidium bromide. DNA of HSV-1 infected cells was digested with Bam HI and subjected to Southern blotting using a LacZ-specific probe. Arrows indicate the specific bands for LacZ and γ_1_34.5 genes.

To determine the presence of the LacZ gene inserted into the deleted site of the γ_1_34.5 gene of R849, the PCR was performed. A LacZ-specific band of 320 bp was detected in R849, RH1 and RH2, but not in HF. When DNA extracted from HSV-1-infected cells was subjected to a Southern blot analysis using the LacZ gene as a probe, a LacZ-specific band was detected in R849, RH1 and RH2; the density in R849 and RH2 was similar, but that in RH1 was reduced by approximately half (Figure 3). In the PCR analysis for the γ_1_34.5 gene, a specific 132-bp band was detected in HF and RH1, but not in R849 and RH2 (Figure 3).

### Formation of plaques by RH1 and RH2 in SAS cells

**Figure 4:**
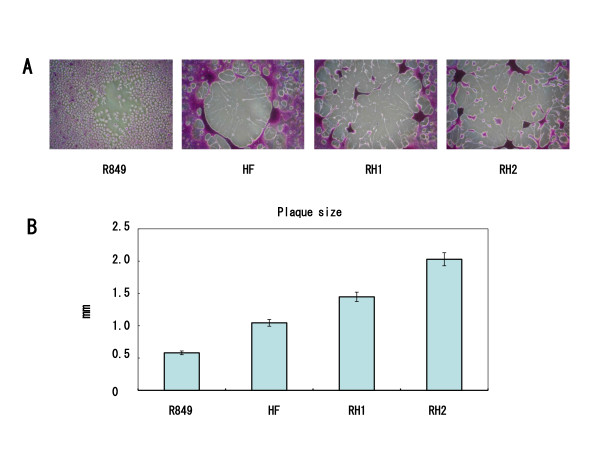
**The plaque size of HSV-1 in oral SCC cells**. (A) SAS cells infected with R849, HF, RH1 and RH2 were incubated for 48 h, and the cytopathic effect was observed. (B) The plaque size was measured. Data are means ± SD of three determinations.

The fusogenic ability of HSV-1 strains was examined by plaque-forming assays in oral SCC cells. SAS infected with R849, HF, RH1 and RH2 were incubated for 48 h, and the cytopathic effect was observed. In the cells infected with RH1 and RH2, extensive cell fusion was observed (Figure 4A). Mean plaque size was greater in RH1 and RH2 than that in HF (RH2 > RH1 > HF); a 2-fold increase was observed in RH2 as compared with HF (Figure 4B). There was a significant (P < 0.05) difference among virus strains.

### Production of RH1 and RH2 in SAS cells

**Figure 5:**
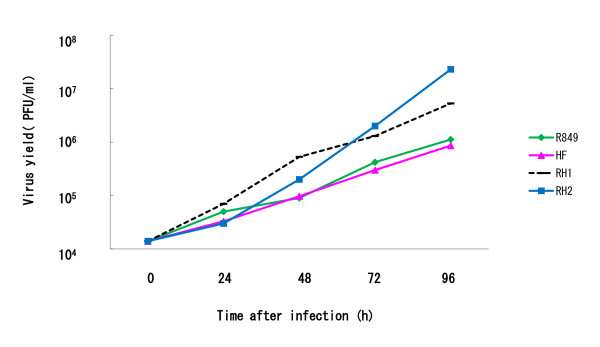
**Replication of HSV-1 in human oral SCC cells**. SAS cells were infected with R849, HF, RH1 or RH2 at a MOI of 0.01 and virus yield was determined at various intervals. Data are means of two determinations. Experiments were repeated three times. A representative result is shown.

The ability of oral SCC cells to support the replication of HSV-1 strains was examined. SAS cells were infected with R849, HF, RH1 or RH2 at a MOI of 0.01 and the virus production was determined at 48, 72, and 96 h after infection. RH1 and RH2 produced more infectious virus than the parental viruses. At 96 h after infection, as compared with the yield of HF, there were 4.2-fold and 23.8-fold increases in those infected with RH1 and RH2 (Figure 5).

### Suppression of the viability of oral SCC cells by RH and RH2

**Figure 6:**
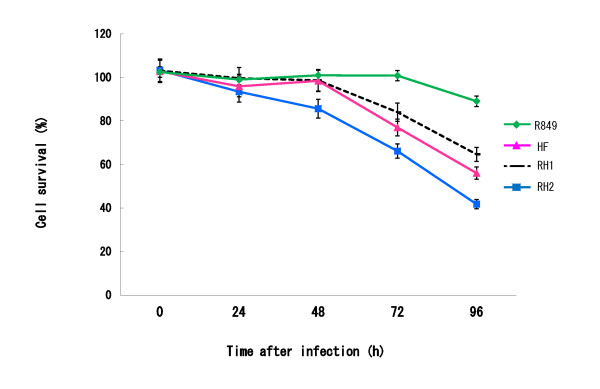
**Suppressive effect of HSV-1 on the viability of oral SCC cells**. SAS cells were infected with R849, HF, RH1, or RH2 at an MOI of 0.01 and cultured at 37°C. Cell viability was measured by the MTT assay. Data are means ± SD of four determinations. Significantly different between HF and RH2 (P < 0.05).

The MTT assay measures the activity of mitochondria. When SAS cells infected with R849, HF, RH1 or RH2 at an MOI of 0.01 were cultured for 96 h, the values were decreased to 88, 54, 64 and 38% of the untreated control, respectively (Figure 6). There was a significant (P < 0.05) difference between HF and RH2.

### Histopathology of HSV-1-injected tumors

**Figure 7:**
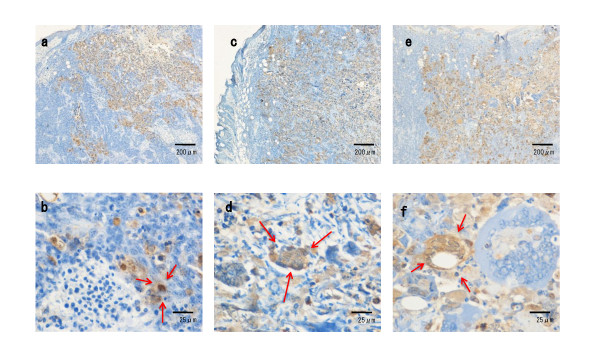
**Histopathological changes and virus gene expression in oral SCC xenografts in nude mice**. Oral SCC xenografts in nude mice were given 1 × 10^6 ^PFUs of R849 (a, b), HF (c, d), or RH2 (e, f). Tumors with skin were removed 3 days after infection and subjected to immunohistochemical staining using antibody against HSV-1. Arrows indicate rounded cells (b) and multinucleated cells (d, f).

**Figure 8:**
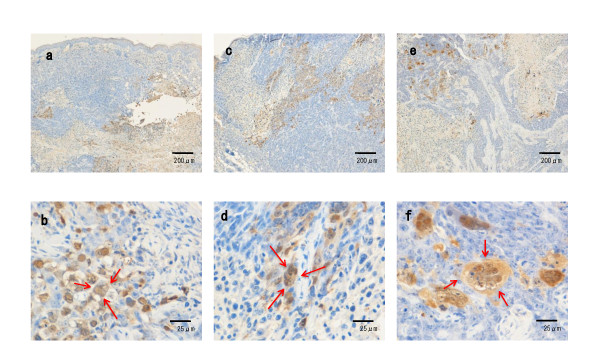
**Histopathological changes and virus gene expression in oral SCC xenografts in nude mice**. Oral SCC xenografts in nude mice were given 1 × 10^6 ^PFUs of R849 (a, b), HF (c, d), or RH2 (e, f). Tumors with skin were removed 7 days after infection and processed as described in Figure 7. Arrows indicate rounded cells (b) and multinucleated cells (d, f).

**Figure 9:**
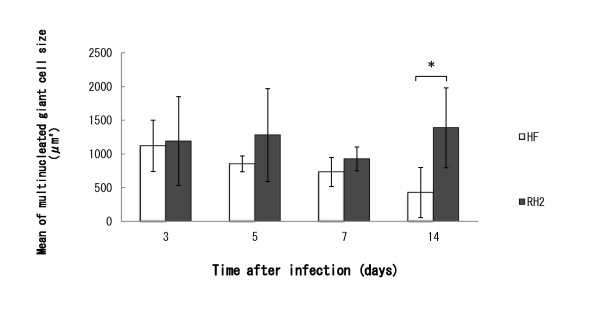
**Histopathological changes and virus gene expression in oral SCC xenografts in nude mice**. Oral SCC xenografts in nude mice were given 1 × 10^6 ^PFUs of HF or RH2. After 3, 5, 7, and 14 days, they were subjected to immunohistochemical staining and the mean size (μm^2^) of HSV-1 antigen-positive multinucleated cells in the sections was determined. Data are means ± SD of three determinations. At 14 days, a significantly difference was observed between HF and RH2 (*P < 0.05).

To examine the expression of HSV-1 antigens, tumors were injected with R849, HF, or RH2 at a dose of 1 × 10^6 ^PFU and subjected to immunohistochemical staining. Three days after injection, the histological change caused by R849 was cell rounding and HSV-1 antigens appeared in these cells. Multinucleated cells appeared in the tumors that received HF or RH2. Especially, both HSV-1 antigen-positive and- negative multinucleated giant cells were observed in RH2-injected tumors (Figure 7). Seven days after the injection of HSV-1, necrotic parts surrounded by HSV-1 antigen-positive cells spread in the tumors. Most multinucleated cells became positive for HSV-1 staining in RH2-injected tumor (Figure 8). When measured at 14 days, the mean size of multinucleated cells induced by RH2 was significantly greater than that induced by HF (P < 0.05) (Figure 9).

### Viruss yields in oral SCC xenografts in nude mice

**Figure 10:**
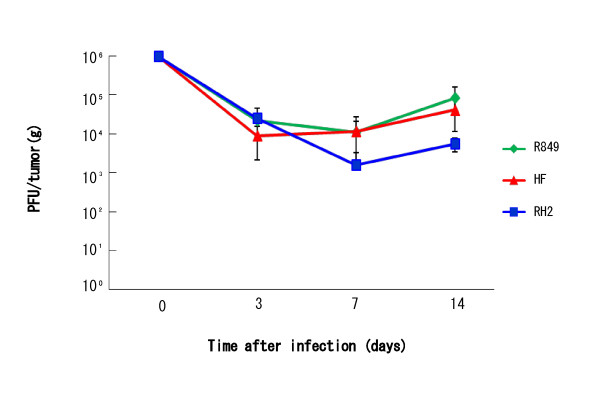
**Virus yields in oral SCC xenografts in nude mice**. Oral SCC xenografts in nude mice were given 1 × 10^6 ^PFU of R849, HF, or RH2 and virus production in the tumors was measured 3, 7, and 14 days after infection. Data are means ± SD of three determinations.

To examine the replication of HSV-1 in vivo, SAS xenografts in nude mice were given 1 × 10^6 ^PFU of R849, HF, or RH2 and virus production in the tumors was examined at 3, 7 and 14 days after injection. The viral titer declined 7 days after injection, but a slight increase was observed 14 days in all strains; the titers in R849, HF and RH2 were 3 × 10^3^, 2 × 10^4 ^and 3 × 10^4 ^PFU/ tumor (g), respectively. There was no significant difference among the strains (Figure 10). The RH2 harvested from tumor tissues at 14 days also showed extensive cell fusion in SAS cells.

### Suppressive effect of HSV-1 infection on the growth of oral SCC xenografts in nude mice

**Figure 11:**
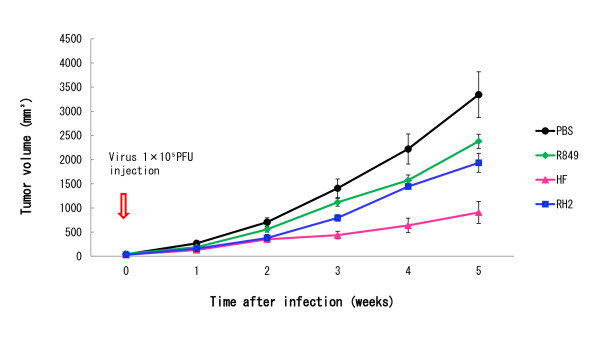
**Effect of R849, HF, and RH2 on the growth of oral SCC xenografts in nude mice**. Tumor-bearing animals received R849, HF, or RH2 at a dose of 1 × 10^5 ^PFU into subcutaneous tumors. Control animals were given PBS. Tumor volume was measured during the experiment. Data are means ± SD of six determinations. Significantly different between control and HF or RH2 (P < 0.05).

Nude mouse tumors were injected subcutaneously with R849, HF, or RH2 at a dose of 1 × 10^5 ^PFU. Control animals were given PBS. In the control group, tumors grew rapidly and the tumor volume at 35 days was 3300 mm^3 ^(Figure 11), whereas the tumor growth of HSV-1-treated animals was suppressed and the tumor volumes were less than 2300 mm^3^. When tumor volume was compared at day 35, a significant difference between the control and HF group or RH2 group (P < 0.05) was found. No symptoms of neurological abnormality and skin reaction at the injected sites were observed during the experiment.

## Discussion

Several viruses that kill their target cells through syncytial formation have been described. The HSV-1 vector Synco-2D has gibbon ape leukemia virus fusogenic membrane protein (GALV-FMG) to causes syncytia to spread in the tumor by cell fusion [14]. Israyelyan et al. [8] reported a syncytial mutation in the gB (Arg to His change at aa 858) and gK (Ala to Val change at aa 40) genes to enhance the fusogenicity of OncdSyn. In this context, a fusogenic HSV-1 under clinical study is HF10. The genome of HF10 has extensive rearrangements at both ends of the UL region and lacks the expression of UL56 and LATs [26]. The lack of UL56 reduces the neuroinvasiveness of HSV without affecting viral replication in vitro. Diakidi-Kosta et al. [27] reported that marker transfer experiments and DNA sequence analysis mapped the syncytial phenotype to a T-C base substitution at codon 787 of the cytoplasmic domain of mature gB, that resulted in Leu to Pro substitution and consequently belonged to the syncytial locus. Indeed, HF10 has an amino acid substitution (Leu to Pro at aa 787) in glycoprotein B (gB) [26], suggesting that the syncytial phenotype of HF10 is ascribed to this amino acid change. HF from which HF10 was cloned has the characteristics of an oncolytic virus. To construct a novel virus that is less neurovirulent with the genetic background of HF, Vero cells were infected with R849 and HF at different MOIs. Since R849 has the LacZ gene inserted into the deleted γ_1_34.5 gene, the expression of LacZ is a marker of a R849-specific structure. We selected fusogenic viruses with the LacZ gene, and obtained RH1 and RH2. When they were subjected to PCR and Southern blot analysis, RH1 and RH2 revealed the presence of the LacZ gene, but the density of the gene was lower in RH1 than in R849 and RH2. Consistent with this finding, the γ_1_34.5 gene was detected in RH1, but not RH2. Thus, it is concluded that one copy of the γ_1_34.5 gene is lost in RH1, whereas both copies are lost in RH2, and that the γ_1_34.5 gene is replaced by a R849-derived DNA fragment with the LacZ gene.

The mean plaque sizes of RH1 and RH2 were significantly greater than that of HF, indicating the ability to induce extensive cell fusion in oral SCC cells. During the simultaneous infection with R849 and HF, a variety of recombinants with a unique phenotype with or without the γ_1_34.5 gene would be produced, although R849 induces cell rounding. Novel mutations in viral glycoprotein genes responsible for extensive cell fusion might occur during or after recombination. It was reported that OncdSyn with mutations at gB and gK caused extensive virus-induced cell fusion in cell cultures, but the titer in tumor cells was a half log lower than that of the wild-type HSV-1 strain F [8]. Synco-2D with GALV-FMG has a significantly increased tumor cell killing ability, but the titer of Synco-2D was also substantially lower than that of the wild-type HSV strain 17 [14]. In the present study, we found that the virus production of RH1 and RH2 was greater than that of the parental viruses. In the MTT assay, RH2 decreased the cell viability most strongly, and RH1 did not exceed the suppressive effect of HF on the growth of tumor cells. Thus, the virus yield in cell culture is not correlated with the ability to induce syncytia and cell death. It can be stated that RH2 has acquired the capability to induce cell fusion and produce virus progeny in oral SCC cells.

Although a number of fusogenic HSV-1 were investigated for their antitumor effect on tumor tissues, the histological alteration associated with HSV-1 infection was not clearly described [8,14,19]. In the present study, R849 induced cell rounding in tumors as in cell culture and viral antigen was demonstrated in rounded cells only. On the other hand, infection with HF or RH2 induced the formation of multinucleated cells. There was variety in size, but RH2 produced larger multinucleated cells than HF, indicating the phenotype in cell culture to be preserved in tumor tissues. We also found multinucleated cells showing only faint staining 3 days after infection with RH2. The viral antigen may be diluted in multinucleated cells, because the infection by RH2 progresses rapidly and involves surrounding uninfected cells. The size of HF-induced multinucleated cells decreased during the observation period, whereas no such alteration was observed in RH2 (Figure 9). This represents the difference in the phenotype of these viruses.

To compare the antitumor ability, we injected R849, HF or RH2 into nude mouse tumors at a low dose. Tumors in control animals increased continuously for 5 weeks, while tumor growth was suppressed by the intratumor injection of HSV-1. A significant difference was demonstrated when HF or RH2 was injected, suggesting that RH2 has antitumor activity like HF. However, the antitumor effect of RH2 was slightly blunted. The virus yields in tumors declined gradually, and a slight increase was observed in 14 days, irrespective of the virus used. Thus, these HSV-1s can replicate in the tumors for at least 14 days. It has been shown that the replication of γ_1_34.5 gene-deficient HSV-1 is dependent on the mitogen-activated protein kinase kinase (MEK) activity and/or phosphoinositide 3-kinase (PI 3-kinase) of the tumor cells [28-30]. This causes the selective replication of the HSV-1 vector including RH2 in tumors. In this respect, HF still retained the γ_1_34.5 gene, so that it can grow in tissues irrespective of MEK and PI 3-kinase activities in the tumor cells. This would explain the difference between HF and RH2 in the antitumor effect and virus yield. It should be also stated that the phenotype of RH2, being favorable for oncolytic virotherapy, was stably maintained after passages in oral SCC cells in culture and nude mouse tumors. We found neither general side-effects nor any neurological disorders in the mice, indicating the low neurovirulence of RH2.

It has been shown that syncytial formation induced by GALV-FMG of Synco-2D can potentiate antitumor immune responses through the increased release of vesicles resembling exosomes [14]. Such structures contain abundant tumor antigens and other molecules involved in antigen presentation (e.g., MHC class I and class II molecules) and can efficiently load dendric cells for cross presentation of tumor antigens [31]. In this regard, intratumoral inoculation of HF10 into mouse cutaneous melanoma inhibited the growth of tumors at both injected and uninjected sites, suggesting the induction of a systemic antitumor immune response by this fusogenic HSV-1 [32]. Thus, RH2 may also enhance tumor antigen presentation by syncytial formation. HF10 is under clinical trials and the genetic structure was published [18-20,26]. It is apparent that the precise structure of the RH2 genome must be determined prior to clinical study.

## Conclusion

This work was conducted to generate a virus that has the syncytial character of HF, while preserving the γ_1_34.5 gene inactivation profile of R849 virus. RH2, a recombinant form of R849 and HF, had a deletion of the γ_1_34.5 gene and exerted a potent cell killing effect on oral SCC cells through a highly fusogenic ability. RH2 may be a candidate for an HF10-based virus vector suitable for brain tumors as well as oral cancer, because of its defect in the neurovirulent γ_1_34.5 gene.

## Competing interests

The authors declare that they have no competing interests.

## Authors' contributions

HT performed half of the experiments, GT performed the rest of the experiments, FO succeeded in the isolation of the recombinants viruses, TI participated in drafting the manuscript, SI participated in the pathological analysis and interpretation of the data, and YY was responsible for the project and for the preparation of the manuscript.

The authors read and approved the manuscript.
